# Operational definitions of paediatric asthma used in epidemiological studies: A systematic review

**DOI:** 10.7189/jogh.11.04032

**Published:** 2021-07-17

**Authors:** Mohammad Shahidul Islam, Samin Huq, Salahuddin Ahmed, Sudipto Roy, Jürgen Schwarze, Aziz Sheikh, Samir K Saha, Steve Cunningham, Harish Nair, RESPIRE collaboration

**Affiliations:** 1 Usher Institute, College of Medicine and Veterinary Medicine, University of Edinburgh, UK; 2 Child Life and Health, University of Edinburgh, UK; 3 Centre for Inflammation Research, University of Edinburgh, UK; 4 Child Health Research Foundation, Dhaka, Bangladesh; 5 Projahnmo Research Foundation, Dhaka, Bangladesh; 6 KEM Hospital Research Centre, Pune, India

## Abstract

**Background:**

Researchers use different definitions to identify children with asthma in epidemiological surveys. We conducted a systematic review to describe the definitions used in epidemiologic studies for wheeze and asthma in the paediatric population, aimed to inform the development of a uniform definition of paediatric asthma for future epidemiological research.

**Methods:**

We systematically searched terms to identify asthma and/or wheeze among children aged <13 years and published between 1995-2020 across seven databases (MEDLINE, EMBASE, PsycINFO, Global Health, AMED, LILACS and CINAHL). PRISMA guidelines were followed for this review.

**Results:**

We extracted a total of 11 886 records, where 190 met our eligibility criteria and included in the analysis. Among the included studies, 62.1% (n = 118/190) used the International Study of Asthma and Allergies in Childhood (ISAAC) questionnaires, predominantly in developing countries (80%, n = 64/80). ‘Wheeze’ was reported in five categories, subdivided by 14 different definitions. “Current wheeze”, defined as caregivers report of wheezing sounds from the chest of the child in the past 12 months and “Wheeze ever”, defined as caregivers’ report of wheezing or whistling in the chest of the child at any previous time, were the most common wheeze category reported in 129 and 95 studies, respectively. Asthma was reported in nine categories using 53 definitions. The most common asthma category was “Asthma ever”, which was reported in 89 studies, based on caregiver statement that the child had asthma in the past.

**Conclusion:**

Definitions of wheeze and asthma for children used in surveys are primarily based on parent-reported clinical features. Studies from developing countries more frequently used the ISAAC definitions to report childhood asthma and wheeze compared to the studies from developed counties. The use of a uniform asthma definition will aid the interpretation of research findings globally.

Asthma is a common chronic lung disease affecting approximately 12% of children aged 6-7 years globally [1] and characterised by narrow bronchial airways causing airway obstruction [2]. The definition and classification of asthma have been continuously reviewed and updated with new information due to the variability of signs and symptoms [3]. Still, an accurate diagnosis of asthma remains a challenge, particularly for children, as it presents different phenotypes depending on the child's age, gender, and ethnicity [4,5].

There is no standard definition of the type, severity, or frequency of symptoms that define childhood asthma [6], making it difficult to ascertain asthma diagnoses using an evidence-based definition. Objective measurements, such as airflow limitation or airway inflammation, the two primary biological parameters of asthma, are difficult to assess in young children as they require patient cooperation [7]. Thus, only a few population-based studies in children have applied objective measurement to identify asthma cases in children [8,9]. Instead, researchers often use operational definitions of asthma, primarily based on parental response to a set of questionnaires developed by the International Study of Asthma and Allergies in Childhood (ISAAC) [9,10]. These operational definitions of childhood asthma directly impact the estimated disease burden and may complicate asthma epidemiology studies. Besides, the use of inconsistent case definitions for asthma surveillance may lead to inaccurate estimations of disease burden, creating difficulty when formulating appropriate asthma prevention policies [11]. Development of a standardised and well-accepted case definition of asthma with high sensitivity and specificity would help in achieving accurate determination of disease burden, international comparisons and formulation of effective prevention and disease control policy.

Therefore, we systematically interrogated the operational definitions of childhood asthma used in epidemiological studies to inform the development of a uniform definition of paediatric asthma for future epidemiological research.

## METHODS

We conducted this review following the Arksey and O'Malley five-stage framework, including identifying the research question, identifying the relevant studies, study selection, data extraction, and collation, summarising and reporting the results [12]. Our research question for this review was: “what are the different case definitions for the diagnosis of childhood asthma that were used in epidemiological studies?”

We identified the relevant studies by searching seven electronic databases: MEDLINE (Ovid), EMBASE (Ovid), PsycINFO (Ovid), Global Health (Ovid), AMED (Ovid), LILACS and CINAHL (EBSCO). We also reviewed the citations of the included studies to ensure that all relevant studies were included.

### Eligibility criteria & search strategies

We explored research articles mainly in two domains: i) articles reporting asthma and/or wheeze, and ii) articles on children. During database searching, we looked for observational studies published between 1 January 1995 and 31 December 2020. We iteratively improved the search strategy by adding variations and equivalents of the keyword “asthma” and “children. Table S1 in the **Online Supplementary Document** provides search terms and searches outcomes.

Study selection and assessment of the methodological quality

We followed the PRISMA guidelines for study selection [13]. We removed the duplicates in our database searches, and three reviewers (SA, SH and MSI) reviewed the titles and abstracts of articles identified. We subsequently reviewed the full-texts of selected articles and extracted data from studies meeting our eligibility criteria. Two reviewers (MSI and SA) independently checked the full text of the selected articles to determine whether they met all eligibility criteria with a third author arbitrating (SH). If more than one article presented similar outcomes and exposure at a similar age in the same cohort, then only one article that provided more epidemiological information was included in the analysis. The following eligibility criteria were used to include articles of epidemiological studies for review: i) publication year between 1 January 1995 and 31 December 2020, ii) age of the participants below 13 years, iii) asthma as the primary outcome reported, iv) the full text was available in English, v) disease was described among human subjects, and vi) included (sample size) at least one hundred participants. We applied the Newcastle-Ottawa Quality Assessment Scale (NOS) to assess the methodological quality of the selected articles [14].

### Data extraction and analysis

We followed the Methodological Expectations of Cochrane Intervention Reviews (MECIR) guidelines for data collection [15]. We extracted the following information: region, country, settings (rural or urban), study design, age and sex of the participants, year of data collection and reporting, who provided the information, data sources, case definitions used to identify asthma patients, disease condition, number of study participants included in the studies and total number of cases identified under each disease condition. We extracted information for the most recent year from the articles that published data at different time points. We coded any response value in the text (eg, sex, study design, settings, disease condition, etc.). The primary aim of data analysis was to identify the different case definitions used to describe asthma phenotypes. We extracted the precise case definitions used in the studies and grouped them into two categories: i) asthma and ii) wheeze, which was further subcategorised. We estimated the asthma disease prevalence based on the studies' definitions to assess their impact on asthma case identification. We presented the data both in numbers and percentage wherever suitable. We assessed the amount of heterogeneity between the studies assessed using I^2^. Anticipating substantial heterogeneity, we decided to use a random-effects model. We used United Nations Human Development Index 2020 to categorise the countries by economic development [16]. We used Statistics and Data Science (StataCorp LLC, College Station, Texas, USA) version 14 to perform the analysis.

### Risk of bias assessment

We used the Newcastle-Ottawa scale (NOS) to assess the risk of bias (ROB) in the included articles. We provide a maximum score of nine points (4 for ‘Selection, 2 for ‘Comparability’ and 3 for ‘Outcome’) spread across three domains [14]. Scoring was undertaken independently by two reviewers, with a third reviewer resolving any disagreements. Studies were considered at low ROB when the overall scores were 8-9; medium ROB when scores were 6-7; and high when the ROB scores were 0-5.

### Role of the funding source

The funder had no role in study design, data collection, analysis and interpretation of data, report writing, or the decision-making process to submit the paper for publication.

## RESULTS

**Figure 1:**
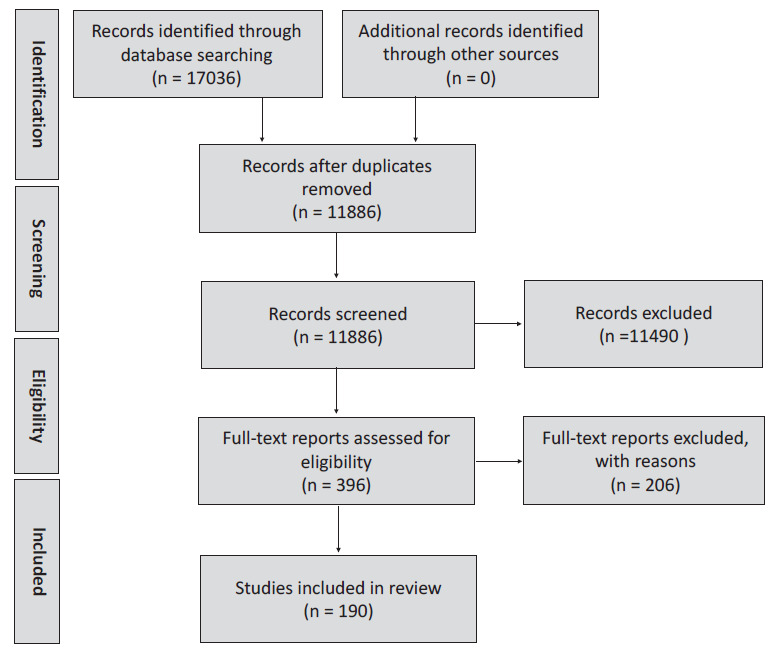
Study selection process for reviewing the definitions of paediatric asthma used in epidemiological studies.

**Figure 2:**
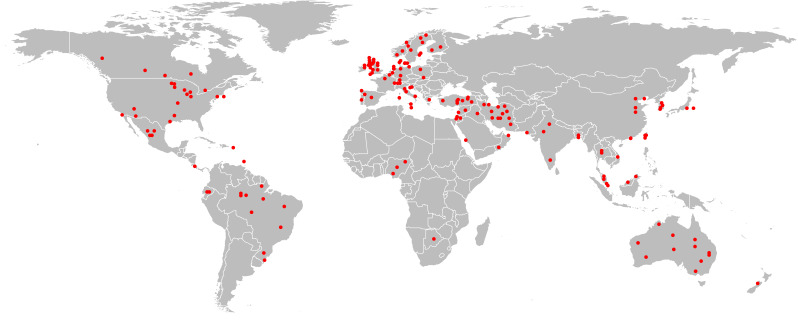
Geographical location of data collection for the selected studies used to review paediatric asthma definitions.

We included 190 articles from 190 different studies in this systematic review (**Figure 1**, Table S2 in the **Online Supplementary Document**). One hundred and three studies (54.2%) included urban population, 23 studies (12.1%) included rural populations, and 64 studies (36.7%) included both urban and rural populations (**Table 1**). Although the selected studies represent all the major geographic regions, the majority were from the European region (n = 74, 39.4%), followed by the Americas region (n = 43, 22.9%) (**Table 1**, **Figure 2**). Among the 190 studies, 158 studies were cross-sectional studies, and 17 were birth-cohort studies (**Table 1**). One hundred and eighteen studies (62.1%) used ISAAC questionnaires, and 58 studies (30.5%) used study-specific questionnaires, and the remaining 14 studies (7.4%) studies used electronic medical records and other sources (**Table 1**). ISAAC questionnaires were used in 80.0% of studies (n = 64/80) conducted in developing countries, compared to only 49.1% (n = 54/110) studies from the developed part of the world. Only three studies have added a pulmonary function test in their asthma definitions [17,18]. Ninety-three percent (n = 177/190) studies collected information from caregivers, and 73.7% (n = 140/190) studies included 6-12 years age children (**Table 1**). Intra-study ROB ranged from 5 to 9, with a mean score of 7.2, showing a generally low risk of bias across the studies. The mean scores across each domain of NOS scale were: 1.6 out of a possible score of 2 points for ‘comparability’; 3.8 out of 5 for ‘selection’; and 1.6 out of 3 for ‘outcome.’

**Table 1:** Characteristics of the selected studies included in the systemic review of the operational definitions of paediatric asthma used in epidemiological studies (n = 190)

Study characteristics	Number of studies (%)
Settings:	
Rural	23 (12.1)
Urban	103 (54.2)
Both	64 (33.7)
Geographic regions:	
African Region	5 (2.7)
Eastern Mediterranean Region	25 (13.1)
European Region	75 (39.5)
Region of the Americas	43 (22.6)
South-East Asia Region	7 (3.7)
Western Pacific Region	35 (18.4)
Country economic status:	
Developing economies	80 (42.1)
Developed economies	110 (47.9)
Type of studies:	
Cross-sectional/prevalence study	158 (83.2)
Retrospective cohort study	6 (3.2)
Longitudinal cohort study	4 (2.1)
Nested cohort study	3 (1.6)
Prospective birth cohort study	17 (9.0)
Randomized controlled trial	1 (0.5)
Case-control study	1 (0.5)
Data source:	
ISAAC standardized questionnaire	118 (62.1)
Study-specific Questionnaire	58 (30.5)
Medical records	6 (3.2)
Health insurance claims	1 (0.5)
Web-based Survey	1 (0.5)
Telephone survey	3 (1.6)
Interview	1 (0.5)
Mixed (questionnaire & clinical assessment)	2 (1.1)
Respondent types:	
Mother/parents	177 (93.2)
Children	4 (2.1)
Studyteam Members	4 (2.1)
Unknown	5 (2.6)
Age categories (years):	
0-5	26 (13.6)
6-12	140 (73.7)
0-12	24 (12.6)

ISAAC – The International Study of Asthma and Allergies in Childhood

### Operational definitions of asthma

Asthma prevalence was reported in two broad groups: wheeze and asthma (**Table 2**). Wheeze was reported in 146 articles under five distinct categories: “Wheeze ever”, “Current wheeze”, “Exercise-induced wheeze”, “Persistent wheezer”, and “Infant wheezer” (**Table 2**). “Wheeze ever” was reported in 95 articles when the children had a wheezing history. For this category, wheezing or whistling in the chest at any time in the past was used in 94 articles (Table S3 in the **Online Supplementary Document**) [19-23]. Children with a wheezing history in the previous 12 months were categorised as “Current wheeze” in 129 articles using eight definitions based on the frequency of wheezing episodes and additional symptoms (Table S3 in the **Online Supplementary Document**) [24-27]. “Exercise-induced wheeze” was reported in 49 articles using the same criterion (children who developed wheeze after exercise) (Table S3 in the **Online Supplementary Document**) [28-31]. Two articles used “Persistent wheezing” for children who had repeated wheeze episodes for an extended period (Table S3 in the **Online Supplementary Document**) [32]. Children aged less than two with a wheezing history were categorised as “Infant wheezers” in one article (Table S3 in the **Online Supplementary Document**) [33].

**Table 2:** Number of identified definitions used to define different type of asthma and wheeze in epidemiological studies

Category and sub-categories	Number of identified definitions	Number of studies reported (%)
Wheeze:		
Wheeze ever	2	95 (50)
Current wheeze	8	129 (67.9)
Exercise-induced wheeze	1	49 (25.8)
Persistent wheezing	2	2 (1.0)
Infant wheezer	1	1 (0.5)
Asthma:		
Asthma ever	10	89 (46.8)
Current asthma	25	55 (28.9)
Doctor-diagnosed asthma	5	76 (40.0)
Diagnosed-asthma	2	2 (1.1)
Asthma-like syndrome	4	3 (1.6)
Probable asthma	3	3 (1.6)
Past asthmatics	2	2 (1.0)
Persistent asthma	2	2 (1.0)
Possible asthma	1	1 (0.5)

Asthma was reported in 180 articles in nine categories (**Table 2**). Children with a history of asthma were reported as “Asthma ever” in 89 articles. We found ten definitions for “Asthma ever”, primarily based on parental reports, physician diagnosis, or recorded in medical records (Table S4 in the **Online Supplementary Document** [34-38]. Children with a recent episode of an asthma attack were categorised as “Current asthma” in 53 articles, and we found 25 different definitions for this disease condition (Table S4 in the **Online Supplementary Document**) [39-43]. Children who had asthma confirmed by a physician at any previous time point were categorised as “Doctor-diagnosed asthma” in 76 articles, and five different definitions were used for this disease condition (Table S4 in the **Online Supplementary Document**) [44-48]. Children who had asthma determined by objective measurement were categorised as “Diagnosed-asthma” in two articles (Table S4 in the **Online Supplementary Document**) [49]. Children with respiratory symptoms similar to asthma were categorised as “Asthma-like syndrome” in five articles with five different definitions (Table S4 in the **Online Supplementary Document**) [50,51]. Children with asthma signs or symptoms which was not confirmed by a physician were categorised as “Probable Asthma” in three articles using three different definitions (Table S4 in the **Online Supplementary Document**) [52]. Children with a history of asthma with no symptoms during the last 12 months were defined as “Past asthma” in two articles using two definitions (Table S4 in the **Online Supplementary Document**) [53]. “Persistent asthma” was reported in two articles for those children who had frequent asthma attacks for a longer period (Table S3 in the **Online Supplementary Document**) [54].

### Regional variation in reporting asthma

We found a significant difference in the reporting of asthma disease prevalence in different World Health Organisation regions. “Wheeze ever” was reported in 60% of articles published in the African region (n = 3/5), compared to 76% (n = 19/25) in the Eastern Mediterranean region, 57.1% (n = 20/35) in the Western Pacific region, 57% (n = 4/7) in the South-East Asian region, 42.6% (n = 32/75) in the European region, and 39.5% (n = 17/43) in the Americas region.

“Current wheeze” was reported in 80% (n = 4/5) articles from African region, 72.0% (n = 18/25) from the Eastern Mediterranean region, 82.9% (n = 29/35) from the Western Pacific region, 57.1% (n = 4/7) from the South-East Asian region, 65.3% (n = 49/75) from the European region, and 58.1% (n = 25/43) from the Americas region.

“Asthma ever” was reported in 40% (n = 2/5) studies from African region, 60.0% (n = 15/25) from the Eastern Mediterranean region, 45.7% (n = 16/35) from the Western Pacific region, 29.0% (n = 2/7) from the South-East Asian region, 45.1% (n = 34/75) from the European region, and 46.5% (n = 20/43) from the Americas region.

Globally, only 28.9% of the selected studies reported “Current asthma” predominantly from the European Region (42.6%, n = 32/75). Still, only in 8.0% (n = 2/25) of the studies from the Eastern Mediterranean region, 25.7% (n = 9/35) studies from the Western Pacific region, 28.0% (n = 2/7) from the South-East Asian region, 23.3% (n = 10/43) from the Americas region reported this disease condition. None of the five articles published in the Africa region reported “Current asthma”.

“Doctor-diagnosed asthma” was reported in 40.0% (n = 76/190) of studies: in 80.0% (n = 4/5) of studies from Africa region, 40.0% (n = 10/25) from the Eastern Mediterranean region, 45.7% (n = 16/35) from the Western Pacific region, 28.5% (n = 2/7) from the South-East Asian region, 40.0% (n = 30/75) from the European region, and 32.5% (n = 14/43) from the Americas region. Other asthma categories were reported in less than 5% of studies, mostly from the studies conducted in Europe and the Americas regions.

### Asthma prevalence by definitions

**Figure 3:**
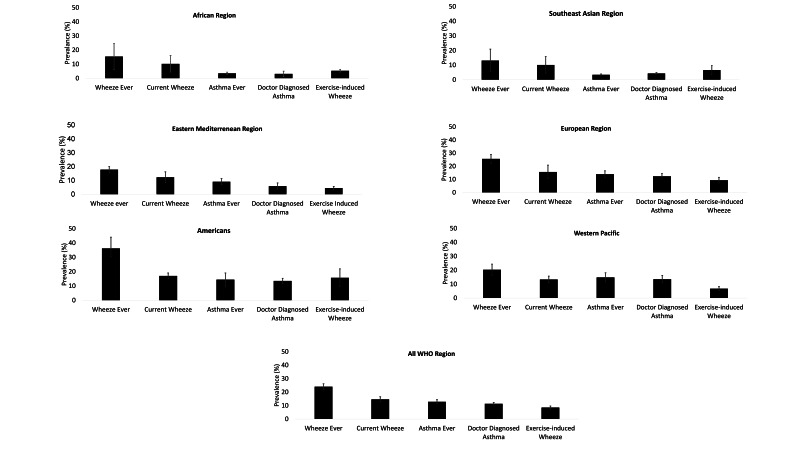
Regional distribution of asthma prevalence in children aged <13 years.

We found “Wheeze ever” as the most prevalent asthma disease category across all regions (**Figure 3**). Our estimated global prevalence of wheeze ever was 24.0% (95% confidence interval (CI) = 21.9-26.1) and ranged between 13.1% (95% CI = 5.3-20.9) in the South-East Asia region to 36.2% (95% CI = 30.2-44.8) in the Americas region. The overall prevalence of “Current wheeze” was 14.6% (95% CI = 12.7-16.5) and ranging between 10.1% (95% CI = 4.4-15.8) in the South-East Asia region to 17.0% (95% CI = 14.9%-19.0%) in the Americas region. The overall prevalence of “Exercise-induced wheeze” was 8.5% (95% CI = 7.4%-9.6%); the lowest was in the Eastern Mediterranean region (4.5%, 95% CI = 3.3%-5.7%) and highest in the Americans region (15.7%, 95% CI = 9.4%-21.9%).

The overall prevalence of “Asthma ever” was 12.8% (95% CI = 15.3%-20.4%) (**Figure 3**); the lowest being in the South-East Asian region (3.4%, 95% CI = 2.9%-3.9%) and highest in the Western Pacific region (14.8%, 95% CI = 11.5%-18.1%). The prevalence of “Doctor-diagnosed asthma” was 11.3% (95% CI = 10.3%-12.3%), and ranging 3.1% (95% CI = 1.1%-5.0%) in African region to 13.3% (95% CI = 11.7%-15.3%) in the Americas region.

## DISCUSSION

We found extensive research on childhood asthma in the past three decades globally. We analysed 190 articles published between 1995 and 2020, representing all regions of the World Health Organisation (WHO). We found 64 definitions, mostly from developed countries using distinct asthma phenotypes to define various asthma types in children; however, objective information from clinical measurements was incorporated only in very few of these definitions. This could be due to the considerable influence of ISAAC (a global initiative established in 1991 to investigate asthma, rhinitis, and eczema in children) in asthma research over the past three decades. Over 50% of the studies included in this review used ISAAC definitions of asthma. The ISAAC research methodologies for evaluating childhood asthma and wheezing were constructed on questionnaire-based responses from the caregivers. Such preference could be linked with its suitability to conduct research at a low cost and with a limited skilled workforce. Despite some limitations, the ISAAC initiative provides strong evidence for the feasibility of developing an optimal universal definition to diagnose childhood asthma at the community level.

On the contrary, we have identified a wide range of definitions for childhood asthma. The maximum variance was found in the definitions of “Current asthma” and “Current wheeze”. Researchers used 25 distinct definitions for childhood “Current asthma” and eight definitions for “Current wheeze”. This variation was due to the use of different time cut-offs and criteria to evaluate and diagnose current asthma. Disease episode during the past 12 months was used to report “Current asthma” (n = 41/53) and “Current wheeze” (n = 126/129) in most of the studies. Nevertheless, three studies defined “Current wheeze” using a period of 3 years [55,56], and 16 studies reported “Current wheeze” without mentioning a specific time period. We also observed a significant disparity in determining “Asthma ever”, which was largely constructed from the parental statement of their children of ever having asthma, either alone or in combination with diagnosis confirmed by a doctor. However, we have observed a consensus in defining “Wheeze ever”; this condition was unanimously defined based on the parental reporting of wheeze or wheezing sound in the chest of their child.

We found only three studies that used pulmonary function test to diagnose asthma [57-59]. However, different guidelines have emphasised using objective assessments, the limitations in the diagnosis of asthma using medical history and physical examination - as a non-reliable means of excluding other diagnoses or of characterising the status of lung impairment [60-62]. Resource scarcity, lack of available expertise in the community and poor patient compliance might be reasons for the low uptake of objective measurement in asthma surveys [63].

We have also estimated the overall prevalence of different asthma diseases conditions and found that the asthma disease burden changes profoundly based on the diagnostic definition used. The reported prevalence of “Wheeze ever” (24.0%) was about twice as high as the prevalence reported for “Asthma ever” (12.8%). This variance in asthma prevalence has a significant public health implication - since the uptake of policy decisions to control or prevent a particular disease largely depend on existing disease prevalence in the area.

To the best of our knowledge, this is the first attempt to systematically investigate the operational definitions used in epidemiological studies in the context of childhood asthma. We used a robust approach for this review that involved multiple researchers independently identifying studies and compared. All data were extracted at least by two researchers. To be inclusive, we have explored seven relevant databases and included studies published in the last 26 years from all geographic regions. As per the NOS criteria, our included articles had a low risk of bias with a mean score of seven out of nine. A potential limitation of this systematic review is that we had to exclude several articles as some of the study subjects did not meet the age limit for this review. Additionally, we could not assess the impact of individual criteria on disease burden estimates as some definitions were used in very few studies.

This review demonstrates a huge research gap in childhood asthma research by economic development. The majority of the included articles (n = 118, 62%) were published from the European and American regions that mostly represent the developed part of the world. Only 12 (6%) included articles published from South East Asia and African regions, representing most of the least developed countries of the world.

The variety of definitions identified in this systematic review illustrates the challenges of establishing an internationally accepted epidemiological definition to measure childhood asthma. Through this review, we have identified a range of asthma definitions, which primarily relied on parent-reported clinical symptoms. We also found that researchers did not use these definitions systematically, and the same definitions were used differently on many occasions. For example, in some studies, researchers diagnosed asthma based on the caregiver statement of a wheezing episode in the previous 12 months, but that definition was mostly used to report “Current wheeze” in other studies [64,65]. Additionally, some definitions were used in different combinations in different studies [66-69].

During the last few decades, there has been little improvement in asthma outcomes despite significant progress in hospitalisation due to a lack of appropriate disease diagnosis. The diagnosis of asthma based on the clinical features and without objective measurement might result in substantial uncertainties in the disease burden and might boost poor asthma management. A recent review of diagnostic accuracy of respiratory diseases in primary health units demonstrated a poor record for asthma diagnosis among the practitioners. The report also found that up to 74% of asthma cases were over-diagnosed in primary health care centres [70]. Further, a prospective multicentre study in Canada failed to diagnose asthma in 33.1% of adults with physician-diagnosed asthma who were not using daily asthma medications or had medications weaned [71]. Therefore, the Lancet Commission on asthma (2017) recommends deconstructing airway disease into component parts for greater precision in asthma diagnosis through the use of spirometry and other (eg, eosinophil count) measures [72]. We re-emphasise the Lancet Commission recommendations as proper diagnosis of asthma using objective measurements both in children and adults are important for patient care as well as for making strategic decisions to reduce the disease burden. Additionally, we recommend the following items listed in **Box 1** while reporting asthma disease burden.We believe that a global initiative to establish accurate childhood asthma measurements will improve prevalence estimation accuracy in future epidemiological asthma surveys. At present, we are doing a feasibility study of using portable spirometers to measure the pulmonary function of children age less than eight years in resource-poor settings. Successful use of our research methodologies will encourage the global community to include such techniques in future asthma disease survey.

Box 1 **Recommendation for future reporting of asthma disease burden estimation**

- Should demonstrate a minimum set of criteria in any asthma disease burden estimation among children which should include the prevalence of history of recurrent wheeze and asthma.

- Should avoid using customised definition of asthma for disease burden estimation.

- Should not amalgamate the definitions available in various guidelines.

- Should use the validated research methodologies in asthma disease burden estimation

- Should use at least one lung function test to confirm the ongoing asthma.

- Should conduct any clinical assessment by medically trained research staff.

## Additional material

Online Supplementary Document
