# Supplementary material for: Operational definitions of paediatric asthma used in epidemiological studies: A systematic review
Source: J Glob Health. 2021 Jul 17;11:04032. doi: 10.7189/jogh.11.04032 (PMC8285759; doi:10.7189/jogh.11.04032)
Supplement: Online Supplementary Document [file jogh-11-04032-s001.pdf]

Supplementary table 1: Search outcomes from database

**Database: EMBASE**		
	Search term	Number of articles retrieved
**1**	asthma.mp.	289817
**2**	Dyspnea, Paroxysmal/	892
**3**	Respiratory Hypersensitivity/	4827
**4**	reactive airways disease.mp.	140
**5**	1 or 2 or 3 or 4	292761
**6**	preschool.mp.	532568
**7**	children.mp.	1321130
**8**	childhood.mp.	427736
**9**	*Child/	74798
**10**	*Adolescent/	26415
**11**	underage.mp.	2661
**12**	7 or 8 or 9 or 10 or 11	1548566
**13**	Case-Control Studies.mp.	24856
**14**	Prospective Studies.mp.	64017
**15**	Cross-Sectional Studies.mp. or Cross-Sectional Studies/	275531
**16**	observational study.mp.	253426
**17**	Case-Control Studies.mp.	24856
**18**	clinical study.mp. or clinical study/	3941346

**19**	14 or 15 or 16 or 17 or 18	4269123
**20**	Respiratory Sound.mp.	8795
**21**	Nitric Oxide.mp.	262687
**22**	Eosinophils.mp.	43230
**23**	Respiratory Function Tests.mp.	2059
**24**	Biomark*.mp.	472825
**25**	RADIOGRAPHY/ or Radiography.mp.	517056
**26**	DIAGNOSIS/ or Diagnosis.mp.	5239434
**27**	Forced Expiratory Volume.mp. or Forced Expiratory Volume/	76160
**28**	Peak Expiratory Flow Rate.mp.	3213
**29**	skin test.mp. or Skin Tests/	36171
**30**	recurrence.mp.	625280
**31**	wheez*.mp.	34172
**32**	20 or 21 or 22 or 23 or 24 or 25 or 26 or 27 or 28 or 29 or 30 or 31	6568800
**33**	5 and 12 and 19 and 32	11748
**34**	limit 33 to (english language and humans and yr="1995 -2020")	10314
**35**	limit 34 to embase	8225

Database: Medline

	Searches	Results
**1**	asthma.mp.	164734

**2**	Dyspnea, Paroxysmal/	353
**3**	Respiratory Hypersensitivity/	9709
**4**	reactive airways disease.mp.	80
**5**	1 or 2 or 3 or 4	170458
**6**	preschool.mp.	939957
**7**	children.mp.	956483
**8**	childhood.mp.	234983
**9**	*Child/	3415
**10**	*Adolescent/	5547
**11**	underage.mp.	1888
**12**	7 or 8 or 9 or 10 or 11	1092210
**13**	Case-Control Studies.mp.	306961
**14**	Prospective Studies.mp.	593908
**15**	Cross-Sectional Studies.mp. or Cross-Sectional Studies/	363663
**16**	observational study.mp.	133089
**17**	Case-Control Studies.mp.	306961
**18**	clinical study.mp. or clinical study/	52730
**19**	14 or 15 or 16 or 17 or 18	1315581
**20**	Respiratory Sound.mp.	132
**21**	Nitric Oxide.mp.	164064
**22**	Eosinophils.mp.	38759

**23**	Respiratory Function Tests.mp.	47659
**24**	Biomarker*.mp.	551875
**25**	RADIOGRAPHY/ or Radiography.mp.	417978
**26**	DIAGNOSIS/ or Diagnosis.mp.	3471555
**27**	Forced Expiratory Volume.mp. or Forced Expiratory Volume/	34872
**28**	Peak Expiratory Flow Rate.mp.	6822
**29**	skin test.mp. or Skin Tests/	41531
**30**	recurrence.mp.	458657
**31**	wheez*.mp.	12741
**32**	20 or 21 or 22 or 23 or 24 or 25 or 26 or 27 or 28 or 29 or 30 or 31	4688100
**33**	5 and 12 and 19 and 32	3968
**34**	limit 33 to (english language and humans and yr="1994 -2020")	3546
**35**	limit 34 to medline	3546
**Database: PsycINFO (Ovid)**		
**1**	asthma.mp. [mp=title, abstract, heading word, table of contents, key concepts, original title, tests & measures, mesh]	8230
**2**	Dyspnea, Paroxysmal/	0
**3**	Respiratory Hypersensitivity/	0
**4**	reactive airways disease.mp.	4

**5**	1 or 2 or 3 or 4	8232
**6**	preschool.mp.	47012
**7**	children.mp.	544418
**8**	childhood.mp.	214531
**9**	Child.mp. [mp=title, abstract, heading word, table of contents, key concepts, original title, tests & measures, mesh]	461132
**10**	Adolescent.mp. [mp=title, abstract, heading word, table of contents, key concepts, original title, tests & measures, mesh]	394722
**11**	underage.mp.	1743
**12**	7 or 8 or 9 or 10 or 11	1024560
**13**	Case-Control Studies/	0
**14**	Prospective Studies.mp.	36397
**15**	Cross-Sectional Studies.mp. or Cross-Sectional Studies/	61475
**16**	observational study.mp.	7737
**17**	Case-Control Studies/	0
**18**	clinical study.mp. or clinical study/	2970
**19**	14 or 15 or 16 or 17 or 18	104289
**20**	Respiratory Sounds.mp.	150
**21**	Nitric Oxide.mp.	6162
**22**	Eosinophils.mp.	196

**23**	Respiratory Function Tests.mp.	483
**24**	Biomarkers.mp.	21192
**25**	RADIOGRAPHY/ or Radiography.mp.	718
**26**	DIAGNOSIS/ or Diagnosis.mp.	205169
**27**	Forced Expiratory Volume.mp. or Forced Expiratory Volume/	530
**28**	Peak Expiratory Flow Rate.mp.	172
**29**	skin test.mp. or Skin Tests/	99
**30**	recurrence.mp.	17787
**31**	wheezing.mp.	218
**32**	20 or 21 or 22 or 23 or 24 or 25 or 26 or 27 or 28 or 29 or 30 or 31	245627
**33**	5 and 12 and 19 and 32	128
	limit 33 to (english language and humans and yr="1995-2020")	124
**Database: Global health**		
**1**	asthma.mp.	22639
**2**	Dyspnea, Paroxysmal/	0
**3**	Respiratory Hypersensitivity/	2499
**4**	reactive airways disease.mp.	7
**5**	1 or 2 or 3 or 4	22974
**6**	preschool.mp.	14506
**7**	children.mp.	265434

**8**	childhood.mp.	43912
**9**	*Child/	0
**10**	*Adolescent/	0
**11**	underage.mp.	526
**12**	7 or 8 or 9 or 10 or 11	271084
**13**	Respiratory Sounds.mp.	35
**14**	Nitric Oxide.mp.	22559
**15**	Eosinophils.mp.	4149
**16**	Respiratory Function Tests.mp.	33
**17**	Biomark*.mp.	44265
**18**	RADIOGRAPHY/ or Radiography.mp.	6694
**19**	DIAGNOSIS/ or Diagnosis.mp.	207017
**20**	Forced Expiratory Volume.mp. or Forced Expiratory Volume/	2198
**21**	Peak Expiratory Flow Rate.mp.	274
**22**	skin test.mp. or Skin Tests/	4035
**23**	recurrence.mp.	22901
**24**	wheez*.mp.	3717
**25**	13 or 14 or 15 or 16 or 17 or 18 or 19 or 20 or 21 or 22 or 23 or 24	295371
**26**	5 and 12 and 25	3449
**27**	limit 26 to (english language and yr="1995 -2020")	3145

**Database: Ahmed**		
**1**	asthma.mp.	2079
**2**	Dyspnea, Paroxysmal/	0
**3**	Respiratory Hypersensitivity/	31
**4**	reactive airways disease.mp.	0
**5**	1 or 2 or 3 or 4	2102
**6**	preschool.mp.	1954
**7**	children.mp.	16011
**8**	childhood.mp.	1836
**9**	Child.mp. [mp=abstract, heading words, title]	21220
**10**	Adolescent.mp. [mp=abstract, heading words, title]	6749
**11**	underage.mp.	4
**12**	7 or 8 or 9 or 10 or 11	28402
**13**	Respiratory Sounds.mp.	52
**14**	Nitric Oxide.mp.	1479
**15**	Eosinophils.mp.	79
**16**	Respiratory Function Tests.mp.	792
**17**	Biomark*.mp.	541
**18**	RADIOGRAPHY/ or Radiography.mp.	2791
**19**	DIAGNOSIS/ or Diagnosis.mp.	25518
**20**	Forced Expiratory Volume.mp. or Forced Expiratory Volume/	377

**21**	Peak Expiratory Flow Rate.mp.	72
**22**	skin test.mp. or Skin Tests/	14
**23**	recurrence.mp.	1577
**24**	wheez*.mp.	93
**25**	13 or 14 or 15 or 16 or 17 or 18 or 19 or 20 or 21 or 22 or 23 or 24	32038
**26**	5 and 12 and 25	86
**27**	5 and 12 and 25	86
**28**	limit 26 to yr="1995 -2020"	74
**29**	limit 27 to English	70
**Database: CINAHL (EBSCO)**		
**S31**	S5 AND S11 AND S17 AND S30	2420
**S30**	S18 OR S19 OR S20 OR S21 OR S22 OR S23 OR S24 OR S25 OR S26 OR S27 OR S28 OR S29	1,181,898
**S29**	Wheezing	2302
**S28**	Recurrence	105,316
**S27**	skin test	19,602
**S26**	Peak Expiratory Flow Rate	2,316
**S25**	Forced Expiratory Volume	9,610
**S24**	Diagnosis	929893
**S23**	Radiography	154043
S22		
	**Biomarkers**	**56806**

**S21**	Respiratory Function Tests.mp.	20434
**S20**	Eosinophils	4240
**S19**	Nitric Oxide	17596
**S18**	Respiratory Sounds	3436
**S17**	S12 or S13 or S14 or S15 or S16	1330458
**S16**	clinical study	741350
**S15**	observational study	81223
**S14**	Cross-Sectional Studies	238624
**S13**	Prospective Studies	522779
**S12**	Case-Control Studies/	96421
**S11**	S6 OR S7 OR S8 OR S9 OR S10	872432
**S10**	MW underage	1
**S9**	(MH "Child, Preschool")	215660
**S8**	Childhood	280344
**S7**	Adolescents	145540
**S6**	Children	753457
**S5**	S1 OR S2 OR S3 OR S4	47106
**S4**	(MH "Respiratory Hypersensitivity")	806
**S3**	(MH "Dyspnea, Paroxysmal")	60
**S2**	reactive airways disease	371
**S1**	Asthma	46414
**Database: Lilacs**		
**1**	**(tw:(((tw:(asthma)) OR (tw:(dyspnea, paroxysmal)) OR (tw:(respiratory hypersensitivity)) OR**	36

(tw:(reactive airways disease))) AND ((tw:(child)) OR (tw:(underage)) OR (tw:(childhood)) OR (tw:(children)) OR (tw:(preschool)) OR (tw:(adolescent))) AND ((tw:(Respiratory Sounds)) OR (tw:(Nitric Oxide)) OR (tw:(Eosinophils)) OR (tw:(Respiratory Function Tests)) OR (tw:(Biomarkers)) OR (tw:(Radiography)) OR (tw:(DIAGNOSIS)) OR (tw:(Forced Expiratory Volume)) OR (tw:(Forced Expiratory Volume)) OR (tw:(Peak Expiratory Flow Rate)) OR (tw:(skin test)) OR (tw:(Skin Tests)) OR (tw:(recurrence)) OR (tw:(wheezing))) AND ((tw:(Case-Control Studies)) OR (tw:(Prospective Studies)) OR (tw:(Cross-Sectional Studies)) OR (tw:(observational study)) OR (tw:(Case-Control Studies)) OR (tw:(clinical study))) AND (la:("en")))) AND ((year_cluster:(1994)) OR (year_cluster:(1995)) OR (year_cluster:(1996)) OR (year_cluster:(1997)) OR (year_cluster:(1998)) OR (year_cluster:(1999)) OR (year_cluster:(2000)) OR (year_cluster:(2001)) OR (year_cluster:(2002)) OR (year_cluster:(2003)) OR (year_cluster:(2004)) OR (year_cluster:(2005)) OR (year_cluster:(2006)) OR (year_cluster:(2007)) OR (year_cluster:(2008)) OR

(year_cluster:(2009)) OR (year_cluster:(2010)) OR (year_cluster:(2011))OR (year_cluster:(2012)) OR (year_cluster:(2013)) OR (year_cluster:(2014)) OR (year_cluster:(2015)) OR (year_cluster:(2016)) OR (year_cluster:(2017)) OR (year_cluster:(2018))) AND (db:("LILACS"))

Supplementary Table 2: List of the included articles

SI	Author	Title	Journal	Year of Publication	Country	Study type	Study population
1	Aberle et al.	Allergic diseases and atopy among schoolchildren in eastern Croatia	Acta Clinica Croatica	2018	Croatia	Cross-Sectional Study	1687
2	Abuekteish et al.	Prevalence of asthma and wheeze in primary school children in Northern Jordan	Annals of Tropical Paediatrics	1996	Jordan	Cross-Sectional Study	3186
3	Adetoun Mustapha et al.	Prevalence of asthma and respiratory symptoms in children in a low socio-economic status area of Nigeria	International Journal of Tuberculosis & Lung Disease	2013	Nigeria	Cross-Sectional Study	339
4	Adgent et al.	A combined cohort analysis of prenatal exposure to phthalate mixtures and childhood asthma	Environment International	2020	USA	Birth Cohort Study	1481
5	Ahmadiashifar et al.	The prevalence and risk factors of asthma, allergic rhinitis, and eczema in primary school children, zanjan, iran	Journal of Advances in Medical and Biomedical Research	2020	Iran	Cross-Sectional Study	800
6	Al-Dawood et al.	Schoolboys with bronchial asthma in Al-Khobar City, Saudi Arabia: are they at increased risk of school absenteeism?	Journal of Asthma	2002	Saudi Arab	Cross-Sectional Study	965
7	Almqvist et al.	Season of birth, childhood asthma and allergy in a nationwide cohort-mediation through lower respiratory infections	Clinical & Experimental Allergy	2019	Sweden	Retrospective Cohort Study	367583
8	Al-Rawas et al.	Trends in asthma prevalence and severity in Omani schoolchildren: Comparison between ISAAC phases I and III	Respirology	2008	Oman	Cross-Sectional Study	4126
9	Al-Riyami et al.	A relatively high prevalence and severity of asthma, allergic rhinitis and atopic eczema in schoolchildren in the Sultanate of Oman	Respirology	2003	Oman	Cross-Sectional Study	3174

10	Al-Thamiri et al.	Asthma prevalence and severity among primary-school children in Baghdad	Eastern Mediterranean Health Journal	2005	Iraq	Cross-Sectional Study	2889
11	Anthracopoulos et al.	Prevalence of asthma among schoolchildren in Patras, Greece: Four questionnaire surveys during 1978-2003	Archives of Disease in Childhood	2007	Greece	Cross-Sectional Study	2725
12	Arnedo-Pena et al.	Risk factors and prevalence of asthma in schoolchildren in Castellon (Spain): a cross-sectional study	Allergologia et Immunopathologia	2009	Spain	Cross-Sectional Study	4292
13	Arshad et al.	Early life risk factors for current wheeze, asthma, and bronchial hyperresponsiveness at 10 years of age	Chest	2005	UK	Birth Cohort Study	1373
14	Austin et al.	Childhood asthma in the highlands of Scotland - Morbidity and school absence	Scottish Medical Journal	2004	UK	Cross-Sectional Study	2549
15	Babak et al.	Association between asthma and body mass index in children	Iran Journal of Allergy Asthma and Immunology	2005	Iran	Cross-Sectional Study	2413
16	Baççıoğlu et al.	The Prevalence of Allergic Diseases and Associated Risk Factors in School-Age Children and Adults in Erzurum, Turkey	Turk Toraks Dergisi / Turkish Thoracic Journal	2015	Turkey	Cross-Sectional Study	494
17	Backlund et al.	Asthma during the primary school ages - Prevalence, remission and the impact of allergic sensitization	Allergy: European Journal of Allergy and Clinical Immunology	2006	Sweden	Prospective Cohort Study	3395
18	Balekian et al.	Cohort Study of Severe Bronchiolitis during Infancy and Risk of Asthma by Age 5 Years	Journal of Allergy and Clinical Immunology: In Practice	2017	USA	Retrospective Cohort Study	3653
19	Banac et al.	Prevalence of asthma and allergic diseases in Croatian children is increasing: Survey study	Croatian Medical Journal	2004	Croatia	Cross-Sectional Study	1634
20	Barnish et al.	Diverging prevalences and different risk factors for childhood asthma and eczema: a cross-sectional study	BMJ Open	2015	UK	Cross-Sectional Study	3805

21	Barraza-Villarreal et al.	Trends in the prevalence of asthma and other allergic diseases in schoolchildren from Cuernavaca, Mexico	Allergy & Asthma Proceedings	2007	Mexico	Cross-Sectional Study	2633
22	Bayram et al.	The prevalence of asthma and allergic diseases in children of school age in Adana in Southern Turkey	Turkish Journal of Pediatrics	2004	Turkey	Cross-Sectional Study	1669
23	Bechtold et al.	Assessing paediatric asthma occurrence through dispensed prescription data and questionnaires	European Journal of Public Health	2013	Italy	Cross-Sectional Study	7014
24	Bedrad et al.	Mediterranean diet during pregnancy and childhood respiratory and atopic outcomes: Birth cohort study	European Respiratory Journal	2020	UK	Birth Cohort Study	8907
25	Berz et al.	Prevalence and correlates of early onset asthma and wheezing in a healthy birth cohort of 2- to 3-year olds	Journal of Pediatric Psychology	2007	USA	Birth Cohort Study	1158
26	Bisgaard et al.	Interaction between asthma and lung function growth in early life	American Journal of Respiratory and Critical Care Medicine	2012	Denmark	Birth Cohort Study	336
27	Bisgaard et al.	Prevalence of asthma-like symptoms in young children	Pediatric Pulmonology	2007	USA	Cross-Sectional Study	1000
28	Bjerg et al.	Time trends in asthma and wheeze in Swedish children 1996-2006: Prevalence and risk factors by sex	Allergy: European Journal of Allergy and Clinical Immunology	2010	Sweden	Cross-Sectional Study	2585
29	Bolat et al.	Prevalence and risk factors for wheezing and allergic diseases in preschool children: A perspective from the Mediterranean coast of Turkey	Allergologia et Immunopathologia	2017	Turkey	Cross-Sectional Study	396
30	Branco et al.	Asthma prevalence and risk factors in early childhood at Northern Portugal	Revista Portuguesa de Pneumologia	2016	Portugal	Cross-Sectional Study	497
31	Branco et al.	Impact of indoor air pollution in nursery and primary schools on childhood asthma	Science of the Total Environment	2020	Portugal	Cross-Sectional Study	1530

32	Broms et al.	Prevalence and co-occurrence of parentally reported possible asthma and allergic manifestations in pre-school children	BMC Public Health	2013	Sweden	Cross-Sectional Study	4886
33	Brozek et al.	Increasing prevalence of asthma, respiratory symptoms, and allergic diseases: Four repeated surveys from 1993-2014	Respiratory Medicine	2015	Poland	Cross-Sectional Study	1698
34	Brustad et al.	High-dose vitamin D supplementation during pregnancy and asthma in offspring at the age of 6 years	Journal of American Medical Association	2019	Denmark	Cross-Sectional	545
35	Burr et al.	Asthma prevalence in 1973, 1988 and 2003	Thorax	2006	UK	Cross-Sectional Study	1148
36	Cai et al.	Household dampness-related exposures in relation to childhood asthma and rhinitis in China: A multicentre observational study	Environment International	2019	China	Cross-Sectional Study	40010
37	Cai et al.	Associations of household dampness with asthma, allergies, and airway diseases among preschoolers in two cross-sectional studies in Chongqing, China: Repeated surveys in 2010 and 2019	Environment International	2020	China	Cross-Sectional Study	2717
38	Carlsen et al.	Asthma in every fifth child in Oslo, Norway: A 10-year follow up of a birth cohort study	Allergy: European Journal of Allergy and Clinical Immunology	2006	Norway	Birth Cohort Study	1019
39	Cesaroni et al.	Individual and are-based indicators of socioeconomic status and childhood asthma	European Respiratory Journal	2003	Italy	Cross-Sectional Study	3917
40	Chatkin et al.	High prevalence of asthma in preschool children in southern Brazil: A population-based study	Pediatric Pulmonology	2003	Brazil	Nested Cohort Study	981
41	Chhabra et al.	Prevalence of bronchial asthma in schoolchildren in Delhi	Journal of Asthma	1998	India	Cross-Sectional Study	816

42	Chinratanapisit et al.	Prevalence and severity of asthma, rhinoconjunctivitis and eczema in children from the Bangkok area: The Global Asthma Network (GAN) Phase I	Asian Pacific Journal of Allergy and Immunology	2019	Thailand	Cross-Sectional Study	3074
43	Cho et al.	Asthma and allergic diseases in preschool children in Korea: findings from the pilot study of the Korean Surveillance System for Childhood Asthma	Journal of Asthma	2014	Korea	Cross-Sectional Study	1002
44	Christiansen et al.	Current prevalence of asthma-related symptoms in San Diego's predominantly Hispanic inner-city children	Journal of Asthma	1996	USA	Cross-Sectional Study	998
45	Cohen et al.	Seasonality of asthma: a retrospective population study	Pedriatrics	2014	Israel	Retropective Cohort Study linked to Medical Charts	542180
46	Coleman et al.	Asthma, wheeze and cough in 7- to 9-year-old British schoolchildren	Ambulatory Child Health	2001	UK	Cross-Sectional Study	1732
47	Csonka et al.	Wheezing in early life and asthma at school age: Predictors of symptom persistence	Pediatric Allergy and Immunology	2000	Finland	Cross-Sectional Study	1816
48	De Britto et al.	Asthma prevalence in schoolchildren in a city in north-east Brazil	Annals of Tropical Paediatrics	2000	Brazil	Cross-Sectional Study	1410
49	De Cassia Ribeiro-Silva et al.	The prevalence of wheezing and its association with serum zinc concentration in children and adolescents in Brazil	Journal of Trace Elements in Medicine & Biology	2014	Brazil	Cross-Sectional Study	592
50	De Farias et al.	Prevalence of asthma in schoolchildren in Alta Floresta- a municipality in the southeast of the Brazilian Amazon	Revista Brasileira de Epidemiologia	2010	Brazil	Cross-Sectional Study	1072
51	De Jong et al.	Diagnosis of asthma in children: Findings from the Swiss paediatric airway cohort	European Respiratory Journal	2020	Switzerland	Cross-Sectional Study	514

52	De Kok et al.	The rate of respiratory symptoms among primary school children in two Dutch regions	European Journal of Pediatrics	1996	Netherland	Cross-Sectional Study	1123
53	De Korte-De Boer et al.	Stabilizing prevalence trends of eczema, asthma and rhinoconjunctivitis in Dutch schoolchildren (2001-2010)	Allergy: European Journal of Allergy and Clinical Immunology	2015	Netherland	Cross-Sectional Study	723
54	Dell et al.	Asthma and allergic disease prevalence in a diverse sample of Toronto school children: Results from the Toronto child health evaluation questionnaire (T-cheq) study	Canadian Respiratory Journal	2010	Canada	Cross-Sectional Study	5572
55	Delpisheh et al.	Salivary cotinine, doctor-diagnosed asthma and respiratory symptoms in primary schoolchildren	Maternal and Child Health Journal	2008	UK	Cross-Sectional Study	425
56	Del-Rio-Navarro et al.	Asthma prevalence in children living in north Mexico City and a comparison with other Latin American cities and world regions	Allergy & Asthma Proceedings	2006	Mexico	Cross-Sectional Study	3211
57	Del-Rio-Navarro et al.	Global Asthma Network Phase i study in Mexico: Prevalence of asthma symptoms, risk factors and altitude associations-a cross-sectional study	BMJ Open Respiratory Research	2020	Mexico	Cross-Sectional Study	35114
58	Del-Rio-Navarro et al.	The burden of asthma in an inner-city area: A historical review 10 years after Isaac	World Allergy Organization Journal	2020	Mexico	Cross-Sectional Study	2535
59	Demir et al.	Asthma and allergic diseases in school children from 1992 to 2007 with incidence data	Journal of Asthma	2010	Turkey	Cross-Sectional Study	422
60	Den Dekker et al.	Breastfeeding and asthma outcomes at the age of 6 years: The Generation R Study	Pediatric Allergy and Immunology	2016	Netherland	Birth Cohort Study	5675
61	Dombkowski et al.	Prematurity as a Predictor of Childhood Asthma among Low-Income Children	Annals of Epidemiology	2008	USA	Cross-Sectional Study	44955

62	Downs et al.	Asthma and hayfever in Aboriginal and non-Aboriginal children living in non-remote rural towns	Medical Journal of Australia	2001	Australia	Cross-Sectional Study	1499
63	Droste et al.	Lung function measures and their relationship to respiratory symptoms in 7- and 8-year-old children	Pediatric Pulmonology	1999	Belgium	Cross-Sectional Study	402
64	Duhme et al.	Asthma and allergies among children in West and East Germany: A comparison between Munster and Greifswald using the ISAAC phase I protocol	European Respiratory Journal	1998	Germany	Cross-Sectional Study	6271
65	El-Sharif et al.	Asthma prevalence in children living in villages, cities and refugee camps in Palestine	European Respiratory Journal	2002	Palestine	Cross-Sectional Study	3382
66	Fadaizadeh et al.	Prevalence and severity of asthma symptoms in students of Tehran and Rasht: Phase III ISAAC study	Tanaffos	2008	Iran	Cross-Sectional Study	6080
67	Falade et al.	Prevalence and severity of symptoms of asthma, allergic rhinoconjunctivitis, and atopic eczema in 6- to 7-year-old Nigerian primary school children: the international study of asthma and allergies in childhood	Medical Principles & Practice	2004	Nigeria	Cross-Sectional Study	1704
68	Faniran et al.	Prevalence of atopy, asthma symptoms and diagnosis, and the management of asthma: Comparison of an affluent and a non-affluent country	Thorax	1999	Nigeria	Cross-Sectional Study	566
69	Farrokhi et al.	Prevalence and risk factors of asthma and allergic diseases in primary schoolchildren living in Bushehr, Iran: Phase I, III ISAAC protocol	Iranian Journal of Allergy, Asthma and Immunology	2014	Iran	Cross-Sectional Study	1280
70	Farzan et al.	Assessment of respiratory health symptoms and asthma in children near a drying saline lake	Environment Research and Public Health	2019	USA	Cross-Sectional Study	357
71	Ferreira-Magalhaes et al.	Asthma-like symptoms, diagnostic tests, and asthma medication use in children and adolescents: A population-based nationwide survey	Journal of Asthma	2016	Potugal	Cross-Sectional Study	437

72	Fritz et al.	Asthmatic disease among urban preschoolers: An observational study	International Journal of Hygiene and Environmental Health	2004	Germany	Cross-Sectional Study	736
73	Galassi et al.	Changes in prevalence of asthma and allergies among children and adolescents in Italy: 1994-2002	Pediatrics	2006	Italy	Cross-Sectional Study	11287
74	Ghaffari et al.	Prevalence of asthma, allergic rhinitis and eczema in elementary schools in sari (Iran)	Caspian Journal of Internal Medicine	2012	Iran	Cross-Sectional Study	1818
75	Glasgow et al.	Asthma screening as part of a routine school health assessment in the Australian capital territory	Medical Journal of Australia	2001	Australia	Cross-Sectional Study	3643
76	Golshan et al.	Prevalence of asthma and related symptoms in primary school children of Isfahan, Iran, in 1998	Asian Pacific Journal of Allergy and Immunology	2001	Iran	Cross-Sectional Study	3838
77	Gong et al.	Parental socioeconomic status, childhood asthma and medication use - a population-based study	PLOS ONE	2014	Sweden	Retropective Cohort Study linked to Medical Charts	211520
78	Grant et al.	Prevalence and burden of illness for asthma and related symptoms among kindergartners in Chicago public schools	Annals of Allergy, Asthma and Immunology	1999	USA	Cross-Sectional Study	638
79	Greaves et al.	Asthma, atopy, and lung function among racially diverse, poor inner-urban Minneapolis schoolchildren	Environmental Research	2007	USA	Cross-Sectional Study	136
80	Griffiths et al.	Childhood asthma prevalence: Cross-sectional record linkage study comparing parent-reported wheeze with general practitioner-recorded asthma diagnoses from primary care electronic health records in Wales	BMJ Open Respiratory Research	2018	UK	Birth Cohort Study	1211
81	Grize et al.	Trends in prevalence of asthma, allergic rhinitis and atopic dermatitis in 5-7-year old Swiss children from 1992 to 2001	Allergy: European Journal of Allergy	2006	Switzerland	Cross-Sectional Study	1274

			and Clinical Immunology				
82	Haby et al.	Asthma in preschool children: Prevalence and risk factors	Thorax	2001	Australia	Cross-Sectional Study	974
83	Hasnain et al.	Prevalence of asthma and allergic rhinitis among school children of Karachi, Pakistan, 2007	Journal of Asthma	2009	Pakistan	Cross-Sectional Study	954
84	Hermann et al.	Prevalence, severity and determinants of asthma in Danish five-year-olds	Acta Paediatrica, International Journal of Paediatrics	2006	Denmark	Cross-Sectional Study	3052
85	Hesselmar et al.	Asthma in children: Prevalence, treatment, and sensitization	Pediatric Allergy and Immunology	2000	Sweden	Cross-Sectional Study	2481
86	Horak et al.	Prevalence of wheezing and atopic diseases in Austrian schoolchildren in conjunction with urban, rural or farm residence	Wiener klinische Wochenschrift	2014	Australia	Cross-Sectional Study	16019
87	Huang et al.	Home environmental and lifestyle factors associated with asthma, rhinitis and wheeze in children in Beijing, China	Environmental Pollution	2019	China	Cross-Sectional Study	2214
88	Kabesch et al.	Lower prevalence of asthma and atopy in Turkish children living in Germany	European Respiratory Journal	1999	Germany	Cross-Sectional Study	5030
89	Kalmarzi et al.	The prevalence of asthma symptoms in elementary and middle school students in Kurdistan province, the west of Iran	Iran Journal of Pediatrics	2016	Iran	Cross-Sectional Study	1768
90	Kalra et al.	Prevalence and risk factors of asthma and wheeze in school-going children in Lucknow, North India	Indian Pediatrics	2004	India	Cross-Sectional Study	3000
91	Kao et al.	The prevalence, severity and seasonal variations of asthma, rhinitis and eczema in Taiwanese schoolchildren	Pediatric Allergy and Immunology	2005	Taiwan	Cross-Sectional Study	3079

92	Kelly et al.	Clinical significance of cough and wheeze in the diagnosis of asthma	Archives of Disease in Childhood	1996	UK	Cross-Sectional Study	3746
93	Kendirli et al.	Prevalence of childhood allergic diseases in Adana, Southern Turkey	European Journal of Epidemiology	1998	Turkey	Cross-Sectional Study	2334
94	Kiboneka et al.	Prevalence of asthma among school children in Gaborone, Botswana	African health Science	2016	Botswana	Cross-Sectional Study	358
95	Kjaer et al.	The prevalence of allergic diseases in an unselected group of 6-year-old children. The DARC birth cohort study	Pediatric Allergy & Immunology	2008	Denmark	Birth Cohort Study	404
96	Kuehni et al.	Prevalence of wheeze during childhood: Retrospective and prospective assessment	European Respiratory Journal	2000	UK	Cross-Sectional Study	1305
97	Kurukulaaratchy et al.	The prevalence of asthma and wheezing illnesses amongst 10-year-old schoolchildren	Respiratory Medicine	2002	UK	Birth Cohort Study	1373
98	Kutzora et al.	Residential crowding and asthma in preschool children, a cross-sectional study	Allergologia et Immunopathologia	2019	Germany	Cross-Sectional Study	4698
99	Kwon et al.	Changes in the prevalence of childhood asthma in Seoul from 1995 to 2008 and its risk factors	Allergy, Asthma and Immunology Research	2010	South Korea	Cross-Sectional Study	4554
100	Kwong et al.	Increasing prevalence of asthma diagnosis and symptoms in children is confined to mild symptoms	Thorax	2001	UK	Cross-Sectional Study	4810
101	Lang et al.	Severe asthma in childhood: Assessed in 10 year olds in a birth cohort study	Allergy: European Journal of Allergy and Clinical Immunology	2008	Norway	Birth Cohort Study	616
102	Lau et al.	Prevalence and risk factors of childhood asthma, rhinitis and eczema in Hong Kong	Journal of Paediatrics and Child Health	1998	Hong Kong	Cross-Sectional Study	3618

103	Lee et al.	Prevalence of childhood asthma in Korea: International study of asthma and allergies in childhood	Allergy, Asthma and Immunology Research	2010	Korea	Cross-Sectional Study	26829
104	Lee et al.	Asthma prevalence among inner-city Asian American schoolchildren	Public Health Reports	2003	USA	Cross-Sectional Study	570
105	Levesque et al.	Asthma and allergic rhinitis in Quebec children	Canadian Respiratory Journal	2004	Canada	Cross-Sectional Study	1267
106	Liao et al.	Prevalence of asthma, rhinitis, and eczema from ISAAC survey of schoolchildren in central Taiwan	Journal of Asthma	2005	Taiwan	Cross-Sectional Study	7040
107	Linehan et al.	Prevalence of respiratory symptoms, features of asthma, and characteristics associated with respiratory disease, in 6-11 year olds in Manchester	Primary Care Respiratory Journal	2009	UK	Cross-Sectional Study	2414
108	Lopez-Silvarrey-Varela et al.	Prevalence and Geographic Variations in Asthma Symptoms in Children and Adolescents in Galicia (Spain)	Archivos de Bronconeumologia	2011	Spain	Cross-Sectional Study	1405
109	Loyo-Berrios et al.	Childhood asthma prevalence in Northern Puerto Rico, the Rio Grande, and Loiza experience	Journal of Asthma	2006	Puerto Rico	Cross-Sectional Study	1467
110	Maitra et al.	Mode of delivery is not associated with asthma or atopy in childhood	Clinical and Experimental Allergy	2004	UK	Birth Cohort Study	7495
111	Malik et al.	Changing trends in asthma in 9-12 year olds between 1964 and 2009	Archieves of Disease in Childhood	2011	UK	Cross-Sectional Study	1196
112	Marks et al.	Prevention of asthma during the first 5 years of life: a randomized controlled trial	The Journal of Allergy and Clinical Immunology	2006	Astralia	Randomized Controlled Trial	260
113	Martinez et al.	Asthma and wheezing in the first six years of life	New England Journal of Medicine	1995	USA	Prospective Cohort Study	826

114	Martyn et al.	Characterization of the duration from onset of asthma symptoms to asthma disease	Annals of Allergy, Asthma and Immunology	2008	USA	Prospective Cohort Study	839
115	Masjedi et al.	Prevalence and severity of asthma symptoms in children of the Tehran-ISAAC study	Pediatric Asthma, Allergy and Immunology	2004	Iran	Cross-Sectional Study	3015
116	Matos et al.	Overweight, asthma symptoms, atopy and pulmonary function in children of 4-12 years of age: findings from the SCAALA cohort in Salvador, Bahia, Brazil	Public Health Nutrition	2011	Brazil	Cross-Sectional Studies nested in a cohort	1129
117	McCann et al.	The prevalence and management of asthma in primary-aged schoolchildren in the south of England	Health Education Research	2002	UK	Cross-Sectional Study	1732
118	McGill et al.	Asthma in non-inner city Head Start children	Pediatrics	1998	USA	Cross-Sectional Study	2215
119	Mehravar et al.	Prevalence of asthma symptoms in Golestan schoolchildren aged 6-7 and 13-14 years in Northeast Iran	Fronteras en Medicina	2016	Iran	Cross-Sectional Study	778
120	Mirzadeh et al.	Asthma and Environmental Factors in Children	Acta Medica Bulgarica	2016	Iran	Cross-Sectional Study	545
121	Mitchell et al.	Cross-sectional survey of risk factors for asthma in 6-7-year-old children in New Zealand: International study of asthma and allergy in childhood phase three	Journal of Paediatrics and Child Health	2009	New Zealand	Cross-Sectional Study	10873
122	Mo et al.	Analysis of prevalence, triggers, risk factors and the related socio-economic effects of childhood asthma in the Student Lung Health Survey (SLHS) database, Canada 1996	International Journal of Adolescent Medicine and Health	2003	Canada	Cross-Sectional Study	11251
123	Moerman et al.	The prevalence of asthma in Canadian children of South Asian descent	Pediatric Pulmonology	2014	Canada	Cross-Sectional Study	7515

124	Mohammad et al.	International Study of Asthma and Allergies in Childhood: phase three in the Syrian Arab Republic	Eastern Mediterranean Health Journal	2010	Syria	Cross-Sectional Study	5107
125	Mohammadzadeh et al.	The prevalence of asthma, allergic rhinitis and eczema in north of Iran: The International Study of Asthma and Allergies in Childhood (ISAAC)	Iranian Journal of Pediatrics	2008	Iran	Cross-Sectional Study	3044
126	Momas et al.	Prevalence of asthma or respiratory symptoms among children attending primary schools in Paris	Pediatric Pulmonology	1998	France	Cross-Sectional Study	3559
127	Moncayo et al.	Risk factors for atopic and non-atopic asthma in a rural area of Ecuador	Thorax	2010	Ecuador	Cross-Sectional Study	1496
128	Montefort et al.	Increasing prevalence of asthma, allergic rhinitis but not eczema in 5- to 8-yr-old Maltese children (ISAAC)	Pediatric Allergy and Immunology	2009	Malta	Cross-Sectional Study	3816
129	Morikawa et al.	Nationwide survey of the prevalence of wheeze, rhino-conjunctivitis, and eczema among Japanese children in 2015	Allergology International	2019	Japan	Cross-Sectional Study	42582
130	Najafizadeh et al.	Prevalence and severity of asthmatic symptoms in Rasht students: A report from ISAAC study	Tanaffos	2008	Iran	Cross-Sectional Study	3065
131	Nelson et al.	Asthma Prevalence in Low-Income Urban Elementary School Students in St. Louis, 1992 and 2004	Journal of Pediatrics	2009	USA	Cross-Sectional Study	331
132	Neuman et al.	Infant wheeze, comorbidities and school age asthma	Pediatric Allergy and Immunology	2014	sweden	Birth Cohort Study	3251
133	Nga et al.	ISAAC-based asthma and atopic symptoms among Ha Noi school children	Pediatric Allergy and Immunology	2003	Vietnam	Cross-Sectional Study	969
134	Ngui et al.	Prevalence of bronchial asthma among Orang Asli in peninsular Malaysia	Medical Journal of Malaysia	2011	Malaysia	Cross-Sectional Study	549
135	Nordlund et al.	Prevalence of severe childhood asthma according to the WHO	Respiratory Medicine	2014	sweden	Birth Cohort Study	3015

136	Nystad et al.	The prevalence of respiratory symptoms and asthma among school children in three different areas of Norway	Pediatric Allergy and Immunology	1997	Norway	Cross-Sectional Study	2791
137	Ochoa-Aviles et al.	Prevalence and risk factors for asthma, rhinitis, eczema, and atopy among preschool children in an Andean city	PLOS One	2020	Ecuador	Cross-Sectional Study	535
138	Ogasawara et al.	Relationship between maternal communicative/critical health literacy and child's asthma symptoms: Results from a population-based survey in metropolitan Japan	Patient Education and Counseling	2020	Japan	Cross-Sectional Study	2612
139	Olaniyan et al.	The association between ambient NO₂ and PM_{2.5} with the respiratory health of school children residing in informal settlements: A prospective cohort study	Environmental Research	2020	South Africa	Retrospective Cohort Study	476
140	Ones et al.	Prevalence of childhood asthma in Istanbul, Turkey	Allergy: European Journal of Allergy and Clinical Immunology	1997	Turkey	Cross-Sectional Study	2216
141	Oren et al.	Cough during infancy and subsequent childhood asthma	Clinical and Experimental Allergy	2015	USA	Birth Cohort Study	410
142	Peat et al.	Prevalence and severity of childhood asthma and allergic sensitisation in seven climatic regions of New South Wales	Medical Journal of Australia	1995	Australia	Cross-Sectional Study	6388
143	Pedersen et al.	Prevalence of atopic dermatitis, asthma and rhinitis from infancy through adulthood in rural Bangladesh: a population-based, cross-sectional survey	BMJ Open	2020	Bangladesh	Cross-Sectional Study	504
144	Peroni et al.	Prevalence of asthma and respiratory symptoms in childhood in an urban area of north-east Italy	Monaldi Archives for Chest Disease	1998	Italy	Cross-Sectional Study	2091
145	Peroni et al.	Preschool asthma in Italy: prevalence, risk factors and health resource utilization	Respiratory Medicine	2009	Italy	Cross-Sectional Study	1402

146	Pike et al.	Physical activity among children with asthma: Cross-sectional analysis in the UK millennium cohort	Pediatric Pulmonology	2019	UK	Cross-Sectional Study	13681
147	Ponsonby et al.	Cross sectional study of the relation between sibling number and asthma, hay fever, and eczema	Archives of Disease in Childhood	1998	Australia	Cross-Sectional Study	6158
148	Prahl et al.	Prevalence of asthma in Danish children aged 8-10 years	Acta Paediatrica, International Journal of Paediatrics	1997	Denmark	Cross-Sectional Study	774
149	Quah et al.	Prevalence of asthma, rhinitis and eczema among schoolchildren in Kelantan, Malaysia	Acta Paediatrica Japonica (Overseas Edition)	1997	Malaysia	Cross-Sectional Study	3939
150	Quah et al.	Prevalence of wheeze, night cough and doctor-diagnosed asthma in pre-school children in Kota Bharu	Asian Pacific Journal of Allergy and Immunology	2000	Malaysia	Cross-Sectional Study	2878
151	Quah et al.	Prevalence of asthma, eczema and allergic rhinitis: Two surveys, 6 years apart, in Kota Bharu, Malaysia	Respirology	2005	Malaysia	Cross-Sectional Study	3157
152	Remes et al.	Prevalence of asthma at school age: A clinical population-based study in eastern Finland	Acta Paediatrica, International Journal of Paediatrics	1996	Finland	Nested Cohort Study	247
153	Robertson et al.	Asthma prevalence in Melbourne schoolchildren: Have we reached the peak?	Medical Journal of Australia	2004	Australia	Cross-Sectional Study	2968
154	Ronmark et al.	Incidence rates and risk factors for asthma among school children: a 2-year follow-up report from the obstructive lung disease in Northern Sweden (OLIN) studies	Respiratory Medicine	2002	Sweden	Prospective Cohort Study	3247
155	Rosa et al.	Prevalence of asthma in children and adolescents in a city in the Brazilian Amazon region	Jornal Brasileiro de Pneumologia	2009	Brazil	Cross-Sectional Study	1634
156	Samolinski et al.	Prevalence of asthma in children, adolescents and young adults in Poland - Results of the ECAP study. [Polish,	Alergia Astma Immunologia	2009	Poland	Cross-Sectional Study	4510

		English]; Wystepowanie astmy oskrzelowej u dzieci, mlodziezy i mlodych doroslych w Polsce w swietle badania ECAP					
157	Santos et al.	Relevance of the first thousand days of life to the development of wheezing in children aged 6-7 years	Allergologia et immunopathologia	2020	Brazil	Case-Control Study	820
158	Selcuk et al.	Prevalence of asthma and allergic diseases in primary school children in Edirne, Turkey, two surveys 10 years apart	Pediatric Allergy & Immunology	2010	Turkey	Cross-Sectional Study	5735
159	Sennhauser et al.	Prevalence of respiratory symptoms in Swiss children: is bronchial asthma really more prevalent in boys?	Pediatric Pulmonology	1995	Switzerland	Cross-Sectional Study	2884
160	Shakurnia et al.	Prevalence of asthma among schoolchildren in Ahvaz, Islamic republic of Iran; Prevalence de l'asthme chez les eleves d'Ahvaz (Republique islamique d'Iran)	Eastern Mediterranean Health Journal	2010	Iran	Cross-Sectional Study	1482
161	Shalowitz et al.	Asthma Burden in a Citywide, Diverse Sample of Elementary Schoolchildren in Chicago	Ambulatory Pediatrics	2007	USA	Cross-Sectional Study	35276
162	Shamssain	Trends in the prevalence and severity of asthma, rhinitis and atopic eczema in 6- to 7- and 13- to 14-yr-old children from the north-east of England	Pediatric Allergy & Immunology	2007	UK	Cross-Sectional Study	1843
163	Shamssain et al.	Prevalence and severity of asthma, rhinitis, and atopic eczema: The north east study	Archives of Disease in Childhood	1999	UK	Cross-Sectional Study	3000
164	Sharma et al.	Prevalence and risk factors for wheezing in children from rural areas of north India. (Special Issue: The continuing challenge of pediatric asthma. Part III.)	Allergy & Asthma Proceedings	2007	India	Cross-Sectional Study	4128
165	Sole et al.	Prevalence of asthma and related symptoms in school-age children in Sao Paulo, Brazil - International study of asthma and allergies in children (ISAAC)	Journal of Asthma	1999	Brazil	Cross-Sectional Study	3005

166	Soto-Martinez et al.	The current prevalence of asthma, allergic rhinitis, and eczema related symptoms in school-aged children in Costa Rica	Journal of Asthma	2018	Costa Rica	Cross-Sectional Study	1417
167	Tai et al.	Prevalence of asthma symptoms and atopic disorders in preschool children and the trend over a decade	Journal of Asthma	2009	Australia	Cross-Sectional Study	1509
168	Takata et al.	Preterm birth is associated with higher prevalence of wheeze and asthma in a selected population of Japanese children aged three years	Allergologia et immunopathologia	2019	Japan	Cross-Sectional Study	6364
169	Tan et al.	Prevalence of asthma and comorbid allergy symptoms in Singaporean preschoolers	Asian Pacific Journal of Allergy and Immunology	2006	Singaporean	Cross-Sectional Study	7549
170	Thongkham et al.	Prevalence and severity of asthmatic symptoms in Grenadian school children: the Grenada National Asthma Survey	BMJ Open	2015	Grenada	Cross-Sectional Study	1374
171	Tortolero et al.	Prevalence of asthma symptoms in a screened school-age population of poor children in Houston, Texas (1997-1998)	Pediatric Asthma, Allergy and Immunology	2007	USA	Cross-Sectional Study	21852
172	Valle et al.	Prevalence and severity of asthma and related symptoms in 6-to 7-year-old schoolchildren of Rio de Janeiro using of the ISAAC questionnaire by telephone survey	Journal of Asthma	2014	Brazil	Cross-Sectional Study	3216
173	Vuillermin et al.	Asthma among school children in the Barwon region of Victoria	Medical Journal of Australia	2007	Australia	Cross-Sectional Study	5176
174	Waked et al.	Asthma, allergic rhinitis and eczema in 5-12-year-old school children across Lebanon	Public Health	2008	Lebanon	Cross-Sectional Study	3907
175	Wanlapakorn et al.	Prevalence of asthma, level of control and factors associated with asthma control in thai elementary school students in bangkok	Asian Pacific Journal of Allergy and Immunology	2014	Thailand	Cross-Sectional Study	1428
176	Waqar et al.	Prevalence of allergy and asthma in school children of Islamabad, Pakistan	World Applied Sciences Journal	2009	Pakistan	Cross-Sectional Study	207

177	Weber et al.	Prevalence of asthma and allergic disorders in regional, rural, and indigenous children aged 6-8 years in Tasmania	Journal of Asthma	2019	Australia	Cross-Sectional Study	1056
178	Webber et al.	Prevalence of asthma and asthma-like symptoms in inner-city elementary schoolchildren	Pediatric Pulmonology	2002	USA	Cross-Sectional Study	4726
179	Werneck et al.	Prevalence of asthma and other childhood allergies in Brazilian schoolchildren	Journal of Asthma	1999	Brazil	Cross-Sectional Study	2714
180	Whyatt et al.	Asthma in inner-city children at 5-11 years of age and prenatal exposure to phthalates: the Columbia center for children's environmental health cohort	Environmental Health Perspectives	2014	USA	Birth Cohort Study	300
181	Wong et al.	Symptoms of asthma and atopic disorders in preschool children: Prevalence and risk factors	Clinical and Experimental Allergy	2007	Hong Kong	Cross-Sectional Study	3089
182	Xie et al.	United States prevalence of pediatric asthma by environmental tobacco smoke exposure, 2016-2017	Journal of Asthma	2020	USA	Cross-Sectional Study	71811
183	Yang et al.	Prevalence of childhood asthma and control in children assessed in a pilot school-based intervention programme in Singapore	Journal of Paediatrics and Child Health	2007	Singapore	Cross-Sectional Study	2119
184	Yang-Huang et al.	Sociodemographic factors, current asthma and lung function in an urban child population	European Journal of Clinical Investigation	2020	Netherlands	Retrospective Cohort Study	5237
185	Yeh et al.	Prevalence and risk factors for early presentation of asthma among preschool children in Taiwan	Asian Pacific Journal of Allergy and Immunology	2011	Taiwan	Cross-Sectional Study	2037
186	Yolsal et al.	Prevalence of asthma among preschool children in Edirne, Turkey	Asian Pacific Journal of Allergy and Immunology	2007	Turkey	Cross-Sectional Study	873
187	Zainal et al.	Prevalence of asthma-like symptoms and assessment of lung function in schoolchildren born with low birth weight	Singapore Medical Journal	2016	Malaysia	Cross-Sectional Study	553

188	Zaman et al.	Asthma in rural Bangladeshi children	Indian Journal of Pediatrics	2007	Bangladesh	Cross-Sectional Study	1587
189	Zhao et al et al.	Prevalence of childhood asthma, allergic rhinitis and eczema in Urumqi and Beijing	Journal of Paediatrics and Child Health	2000	China	Cross-Sectional Study	5538
190	Zobeiri et al	Prevalence, risk factors and severity of asthma symptoms in children of Kermanshah, Iran: ISAAC phase I, II	Acta Medica Iranica	2011	Iran	Cross-Sectional Study	3036

Supplementary table 3: Frequency distribution of definitions used in different studies to define wheezes

Categories	Criteria	Study numbers
**Wheeze Ever**	Reported wheezing or whistling in the chest at any time in the past	94
	Experienced dyspnea, chest tightness and/or wheezing during the age period 0-3 and/or 4-10 years	1
**Current Wheeze**	Parent-reported wheezing or whistling in the chest in the past 12 months	119
	Reported wheezing or chest noise at any time in the last 12 months	2
	Parent-reported wheezing in the last three years	3
	Reported four or more wheezing episodes in the last 12 months	1
	Parent-reported wheezing on more than one occasion during the past year and no asthma diagnosis or emergency room visit for asthma	1
	Parent-reported whistling or chest cold in the last 12 months	1
	Parent-reported wheezy breathing and dyspnea during the last 12 months	1
	Reported frequent wheezing without cold or flu in the past 12 months	1
**Exercise-Induced Wheeze**	Reported wheezing due to exercise in the last 12 months	49

**Persistent Wheezing**	Reporting of at one illness of the lower respiratory tract alongside wheezing in the first three years of life and subsequent wheezing at six years of age	1
	Reported wheezing for at two different time points	1
**Infant Wheezer**	Reported wheezing during the first two years of life	1

Supplementary table 4: Frequency distribution of the definitions used in different studies to define asthma

**Categories**	**Criteria**	**Studies Number**
**Asthma Ever**	Parent-reported history of asthma	70
	Primarily diagnosed asthma either documented in the medical bills or reported at least two asthma medication events in the last 12 months	3
	Reporting minimum two of the following: a) physician-diagnosed asthma, b) reported dyspnea, chest tightness or wheeze, c) said usage of asthma medication	2
	Physician stated asthma diagnosis in the last two years or the reporting of two or more separate events of wheezing by the physician prior to two years	1
	Reported doctor-diagnosed asthma, asthma bronchial, asthmatic or spastic bronchitis anytime in the past	2

	Parent-reported doctor-diagnosed asthma or emergency room visit for at least once in the past year for an asthmatic episode	1
	Reported history of medically diagnosed asthma	7
	History of wheezy breathing associated with dyspnea	1
	Recurrence of at least two of the three symptoms (cough, wheeze, and shortness of breath) within the past 12 months or asthma diagnosed by the doctor or reported symptoms suggestive of asthma within the previous 12 months in combination with a positive response to objective measures such as bronchodilator or exercise tolerance test	1
	Any of the following: i) doctor-diagnosed asthma, ii) a history of cough, dyspnea and/or wheezing or history of cough, iii) dyspnea plus wheezing on examination and reporting of substantial variability in symptoms, iv) a reporting of two or more of the following: sleep disturbed by nocturnal cough and wheeze, nonsmoker (aged 14 years), nasal polyps, blood eosinophilia (eosinophil count 300/L), a positive response to wheal-and-/flare skin test results or elevated level of serum IgE level, reported history of hay fever or infantile eczema or cough, dyspnea, and wheezing regularly on exposure to an antigen and pulmonary function test results showing Forced expiratory volume in 1 second (FEV1) or forced vital capacity reading of less than 70% of predicted value and another test with at least of 20% improvement of the predicted FEV_1 value of a methacholine challenge test showing ≥20% decrease in FEV_1 value	1
**Current**	Reported wheezing in the last 12 months with diagnosed asthma	15

**Asthma**	Doctor-diagnosed asthma and use of asthma medicine or reported wheezing episodes in the last 12 months	8
	Reported history of asthma with either i) reported dyspnea, chest tightness, wheeze, ii)usage of asthma medications, iii)positive result of the exercise test	2
	Doctor-diagnosed asthma reported during the last 12 months	2
	A history of recurrent wheezing during the past one year or a past of recurrent wheezing and current history of exercise or cold-induced wheezing or contemporary history of exercise-and cold-induced wheezing	1
	Wheezy breathing, emergency room or hospital treatment for acute asthma, or regular anti-inflammatory therapy for chronic asthma	1
	Doctor-diagnosed current asthma	1
	Parent-reported current asthma	4
	Reporting asthma episodes in the last 12 months	3
	Reporting of recurrent periods or attacks of coughing and/or dyspnea with or without wheezing within the preceding year	1
	Reported wheeze in the past 12 months or positive response to ‘ever had asthma’ along with exercise-induced wheeze or night cough in the past 12 months	1
	Reporting of doctor-diagnosed asthma within the last two years or a doctor confirmation of two or more episodes of wheezing in the same periods	1

	Parent-reported wheeze in the previous 12 months and/or diagnosed asthma or >12 increase FEV_1 post-administration of a bronchodilator	1
	Presence of asthmatic symptoms in the last 12 months	1
	Parent-reported asthma at any time in the past associated with wheezing in the last 12 months or wheezing four times or more in the previous 12 months, or physician diagnosis of asthma and current symptoms or current usage of inhalation steroids	1
	Reporting doctor-diagnosed asthma, bronchitis, spastic bronchitis or allergic bronchitis in last one year	1
	Parent-reported ever asthma associated with 1)wheezing, 2) walking with breathlessness, 3) asthma attack in the last 12 months or 4) taking asthma medication at the time of interview	1
	Parent-reported ever asthma associated with 1)wheezing, 2) walking with breathlessness, 3) asthma attack in the last 12 months or 4) taking asthma medication at the time of interview	1
	Reporting of more than one asthma episode in the last 12 months	1
	Occurrence of wheezing in the last 12 months and one of the following: diagnosis of asthma at least once, waking up at night because of wheezing in the past 12 months, wheezing while exercising in the last 12 month and four or more episodes of wheezing	2
	Reporting of at least two of 1) intake of asthma medication due to the presence of asthma symptoms in the last 12 months, 2) history of wheeze in the same period, 3) doctor’s diagnosis of asthma	1

	Wheeze and airway hyperresponsiveness diagnosed by bronchial challenge test using a spirometer	1
	Children diagnosed with asthma at the time of examination by a physician	1
	History of asthma or attack of shortness of breath with wheeze in the past 12 months	1
	Recorded recurrent episodes of troublesome lung symptoms (five events within six months lasting three consecutive days) or presence of typical asthma symptoms or use of inhaled beta 2-agonist, or using a 3-month course of inhaled corticosteroids and relapse when stopping treatment	1
	received asthma medication in follow-up period	1
**Doctor Diagnosed Asthma**	Parent-reported doctor-diagnosed asthma	72
	Asthma diagnosed by the doctor with a history of wheezy breathing and dyspnea	1
	Parent-reported doctor or medically diagnosed asthma	1
	Doctor-diagnosed asthma with a history of wheeze	1
	Reporting doctor-diagnosed asthma alongside current symptoms	1
**Diagnosed Asthma**	Positive response to lung function evaluation through spirometry	1
	At least one reported asthmatic symptom with excessive variability in lung function test	1

**Asthma Like Syndrome**	Presence of cough, whistling sounds in chest, breathlessness or cough, whistling sounds in chest or whistling sounds, breathlessness or presence of only asthma-like cough	1
	At least one of the three symptoms in the last 12 months: wheezing, nocturnal symptoms or asthma attacks (wheezing was further described as wheezing with breathlessness or wheezing without a cold	1
	Positive response to any of the questions on ever having had asthma, current asthma or current wheeze	1
	Repeated episodes (days) with troublesome coughing or troublesome noisy breathing or troublesome breathlessness or shortness of breath or taking medicines, pills, puffers or other medication for troublesome respiratory	
	Cough without wheeze reported for more than three weeks or reported wheeze after playing for at least two different episodes	1
**Probable Asthma**	Parent-reported history of exercise-induced or cold-induced wheezing	1
	Reporting of asthma ever alongside positive responses to other four asthma-related questions in the ISAAC questionnaire	1
	History of wheezing or doctor diagnosis of asthma with an indication of the respiratory symptom(s) within the past year or usage of medication for the relief of respiratory symptoms	1
**Past Asthma**	History of recurrent wheezing in the past but no symptoms during the past one year and no current history of exercise-or cold-induced wheezing	1

	Positive response to ever asthma and Negative response to all other asthma-related questions	1
**Persistent Asthma**	Four or more asthma medication-dispensing events, or at least one visit to the emergency department with a primary diagnosis of asthma, or at least one visit to the hospital with a primary diagnosis of asthma, or at least four outpatient visits with a primary diagnosis of asthma and at least two asthma medication-dispensing events	1
	Frequently disturbing asthmatic attacks resulting in absence from school due to more than four such attacks during the last 12 months	1
**Possible Asthma**	Positive response to two of the following symptoms: breathlessness, chest tightness, mucus production or congestion	1
